# Supplementary material for: Tissue Microarray Lipidomic Imaging Mass Spectrometry Method: Application to the Study of Alcohol-Related White Matter Neurodegeneration
Source: Appl Biosci (Basel). Author manuscript; Available in PMC 2024 Feb 21. (PMC10880182; doi:10.3390/applbiosci2020013)
Supplement: Supplementary Data [file NIHMS1965956-supplement-Supplementary_Data.pdf]

Supplementary Materials: Tissue Microarray Lipidomic Imaging Mass Spectrometry Method: Application to the Study of Alcohol-Related White Matter Neurodegeneration

Isabel Gameiro-Ros [4,†], Lelia Noble [1,†], Ming Tong [2,†], Emine B. Yalcin [1] and Suzanne M. de la Monte [1,2,3,*]

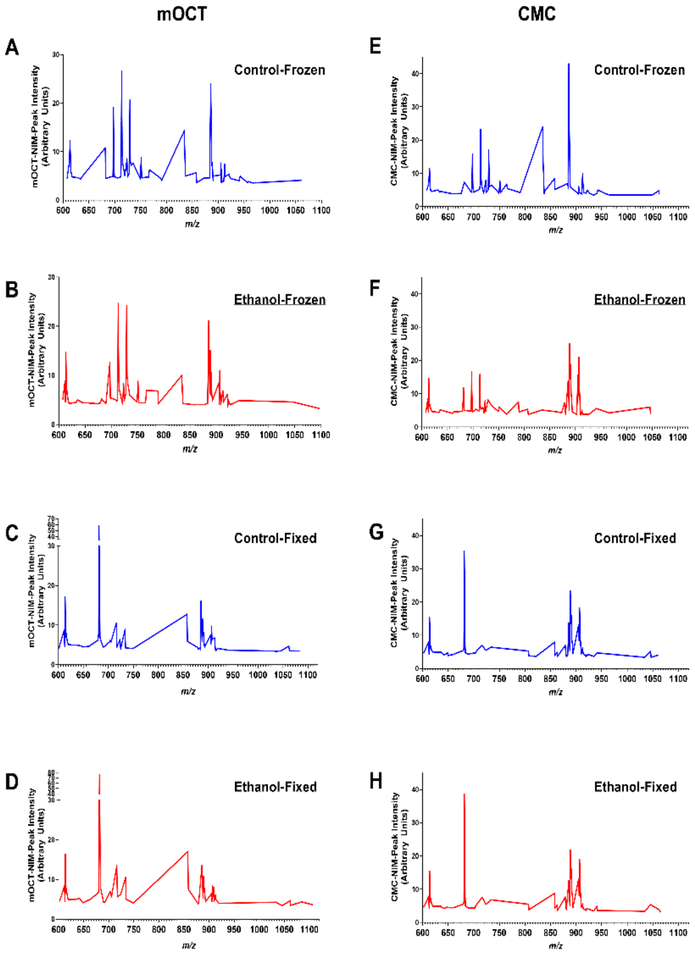

Figure S1. MALDI-IMS spectra corresponding to (A,B,E,F) fresh frozen or (C,D,G,H) formalin-fixed frontal lobe white matter samples from (A,C,E,G) control and (B,D,F,H) ethanol-fed rats. 3-mm tissue cores were embedded in a hybrid (A-D) mOCT/(E-H) 2% CMC TMA and following DHB sublimation coating, the samples were imaged in the negative ion mode (NIM) along with calibration standards used to assess the relative abundance (peak intensity-arbitrary units) of each lipid ion. Each spectrum represents averaged results from 2 rats per group. See Figure S2.

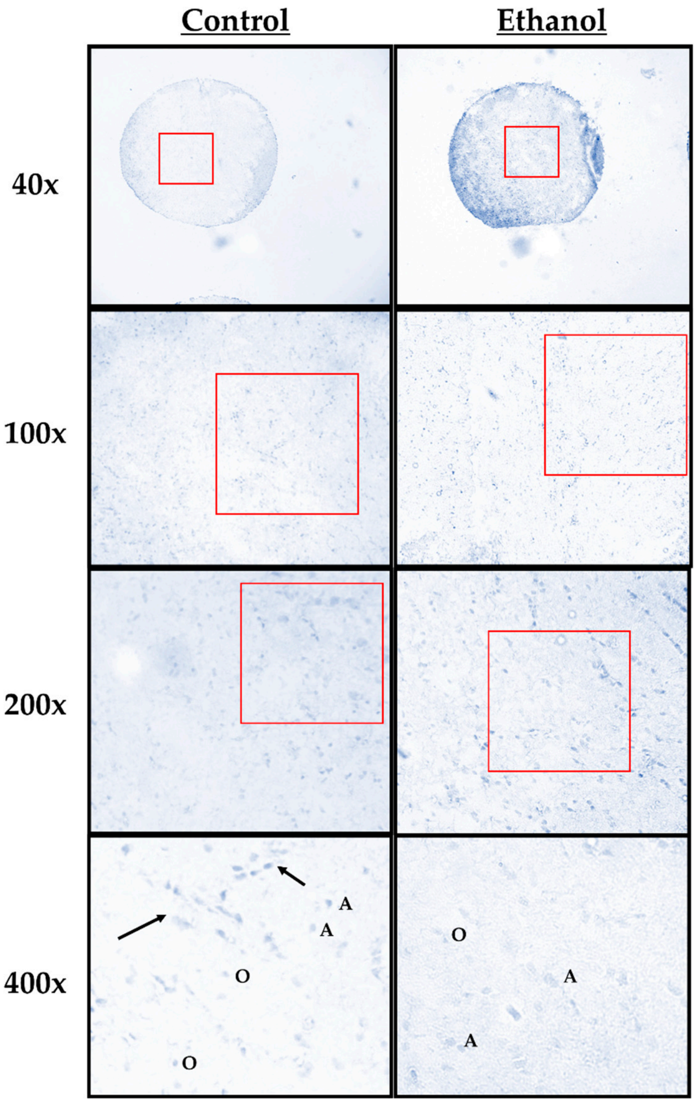

Figure S2. Example optical images of Hematoxylin-stained TMA frontal white matter cores from control and chronic ethanol-fed rats corresponding to the 4th core from the left in each group shown in Figure 6. Hematoxylin labels nuclei. The cores were photographed at different magnifications to reveal the glial-predominant and microvascular composition of white matter. Example oligodendrocytes with dot-like nuclei and astrocytes with oval nuclei are depicted to the left of the O's or A's. Microvessels with narrow lumens are marked with arrows. The red squares show the regions of higher magnification depicted in the immediately below panels.
